# Essays on the Puerperal Fever, and Other Diseases Peculiar to Women; Selected from the Writings of British Authors Previous to the Close of the Eighteenth Century

**Published:** 1850-08

**Authors:** 


*Essays on the Puerperal Fever, and other diseases peculiar to Women; selected from the writings of British Authors previous to the close of the eighteenth century.* Edited by FLEETWOOD CHURCHILL, M. D., M. R. I. A., &c. &c. Philadelphia: Lea & Blanchard, 1850. (*Reprint from the Sydenham Society's last publication.*)

Dr. Churchill's collection of essays, taken from the written experience of seven distinguished physicians, on the subject of puerperal fever, is in every way worthy the respect of the profession. The author has not only given us the various monographs on the disease, commencing with that of Dr. Denman on the sickness of 1768, and terminating with Dr. Gordon's well known essay on the epidemic puerperal fever which occurred at Aberdeen in 1789, but he has greatly enhanced the value of the work by adding to the selected matter a short, condensed, but very interesting historical sketch of the different epidemics of puerperal fever which have at different periods swept off the unfortunate inmates of the various lying-in hospitals of Europe. He commences with a description of the fatal effects of this disease, and the mournful impression produced upon his mind by witnessing its ravages; "a picture," he adds, "whose gloom is heightened by the inutility of all precautions to guard against its attacks, and, in the majority of cases, the utter failure of all attempts to arrest its progress, or to prevent its fatal termination."

Impelled by such feelings, Dr. Churchill has presented to the reader a selection of such works upon the subject as in his judgement is best calculated to afford a complete view of the disease in itself, and especially of the aspect it presents when it occurs epidemically; and not only so, but by selecting descriptions of the various epidemics of England, Ireland and Scotland, the various characters of the disease, and its particular modifications are illustrated by those who witnessed them.

Dr. C. disclaims all intention of compiling a treatise on puerperal fever, his sole object is to collect the principal facts connected with its history as given by persons who witnessed its desolating course, and thereby afford the student and physician of our own time an opportunity of comparing the symptoms, treatment and mortality of the different epidemics, in order, if possible to do away with that one sided view of the disease which persists in asserting that child-bed fever is inflammation and nothing but inflammation, an infatuation which we sincerely hope has nearly satiated itself with human blood.

However, we will not anticipate our author, but suffer him in due time to speak for himself, not having the vanity to suppose, though we have often felt as he feels, that we can write upon the subject with the same force and clearness.

According to our author, the first undoubted epidemic of puerperal fever on record is that which prevailed in Paris during the winter of 1746; it was extremely fatal, attacking the poor, and proving much more fatal to those *in hospital*, than to those who were delivered at their own houses. Of twenty women, in February of that year, in the Hotel Dieu, scarcely one recovered; they died between the 5th and 17th days after their confinement. As the post mortem appearances described afford nothing particularly new to the instructed physician of the present day, we do not think it desirable to consume our necessarily confined space by quoting them.

The treatment of the disease is not given.

After this we have a long list of successive epidemics, as they have occurred in the different lying-in hospitals of Europe, but as they are little more than a list of dates without detail, either of symptoms or of treatment, of course it would be useless, in a general review, to do more than allude to them as a portion of the history, though their perusal, we make no doubt, would greatly interest the enthusiastic student of the result.

The author, however, quotes largely from a M. Tenon of Paris, who says that " two distinct forms of the disease were successively observed in the years 1774–5. One a simple form, which was cured by ipecacuanha," which we should be inclined to suspect was not the disease at all; " the other, a complicated form for which there was no remedy, so that there perished one of every seven of those who were attacked, and death took place from the sixth to the eighth day, and often much earlier.

The following quotation is a good description of the symptoms of the milder cases:

"The first symptoms manifested themselves twenty-four, thirty-six, or forty-eight hours after delivery, and sometimes, but rarely, in the space of twelve hours. The symptoms of the simple puerperal fever are developed in the following order: rigor, slight pain in the kidneys, intestinal colic, which in two hours affects the whole hypogastrium, and gradually becomes more acute, pulse concentrated, fever moderate, lochia not suppressed, mammæ flaccid, tongue dry in the middle, covered with a yellow mucous on the edges, hiccup and vomiting of green colored matter. There was sometimes combined with these constant and characteristic symptoms, a diarrhœa of a bilious glairy matter, a considerable swelling of the hypogastrium, thirst, and remarkable retention of urine.

"In the complicated puerperal fever the pyrexia is more intense, with exacerbations; the tongue is black and dry, the belly is tense, distended and tympanitic and slightly painful. In some women the lochia have been either wholly suppressed or only diminished, others have experienced attacks of ophthalmia, in some the respiration was difficult, in general the blood showed the buffy coat.

"On opening the abdomen, the stomach, and intestines, particularly the small intestines, were inflamed, adhering to one another, distended with air and a yellow fluid matter. The uterus was contracted to its ordinary dimensions, and was seldom inflamed. I had occasion to dissect two; in one, the uterus contained a coagulum of blood; an infiltration of a milky appearance, or whey-like fluid, existed in certain women, in the cellular tissue surrounding the kidneys. Sometimes also a thick white cheesy matter was met with. When the lungs were gorged with blood, or inflamed, or emphysematous, an effusion of serum was found on each side of the chest. We did not observe the hemorrhages which occurred in the epidemic of 1664, and the uterus was not found dry and hard, and tumefied, as in that of 1746. In the epidemic of 1774 the lochia flowed, but they did not flow in 1746."

This valuable description blends together more than one epidemic, but as the disease is stated to have presented the same characters, the author has chosen to quote it in this place.

In the year 1782 the Royal Medical Society of Paris made a report to the French government on M. Doulcet's method of treatment, whose remedy it appears was an emetic of ipecacuanha, followed by a gentle purgative, which is said to have been very successful. This certainly leads us to infer that most of his cases must have been of a very mild character.

From the year 1765 to 1775, puerperal fever appears to have prevailed in Derbyshire and the adjacent counties. Butler describes it, but its course must have been unusually mild, as Dr. Butler states that "ten grains of rhubarb and ten of aromatic confection, given every day until the stools became natural, never failed to effect a cure." He objects to bleeding, very naturally, as the affection he describes must have been comparatively trifling. Dr. Gordon's cases in the epidemic at Aberdeen, 1789, are mentioned, with the treatment, which every body knows was bleeding—prompt and decided bleeding—irrespective of the state of the pulse ; but as Gordon's valuable treatise has been for years in the hands of every practitioner in the United States, and as it is reprinted in the book, we shall make no further comment on it.

"Dr. Gooch has furnished us," says the author, "with the experience of Dr. Lowder, who practiced in London about this time." He, Dr. Lowder, "thought that the inflammation was erysipelatous, and the fever typhoid. When the inflammatory symptoms were well marked, he permitted a few ounces of blood to be drawn, but if the symptoms were typhoid, bleeding was positively injurious, he mentioned it as the assertion of many medical men that *all* the patients who were bled died. When the fever was typhoid, he recommended bark, and mentioned two cases apparently hopeless which recovered by taking daily a gallon of the decoction."

In 1809-10 Hey lost eleven patients out of fourteen, only three recovering ; this was before Mr. Hey adopted Dr. Gordon's plan of taking blood largely at the beginning ; he afterwards by the bloodletting practice saved fourteen out of seventeen. This was certainly being very successful, but we are much inclined to suspect, (though we approve highly of the sanguineous depletion in some cases) that he had to deal with a much less fatal form of the disease than when he saved but three out of fourteen ; medical gentlemen are very fond of parading their successes, but they are not always so ingenuous as to tell us *all* the causes of their success.

We must apologise for the length of this list of epidemics, but though it may weary the mind with its monotony, it has the advantage of showing us what an amount of difference of opinion exists in the treatment of this disease ; how different the various epidemics are in character; and that although large abstractions of blood may in one case be the only means of saving our patient, yet in another, so far from being beneficial, they will only hurry the miserable sufferer to her grave.

The author makes frequent quotations from Dr. Douglas, who has given a good account of the epidemic of 1810–11, as presenting itself in the Dublin Lying-in Hospital.

Dr. Douglas describes three varieties of puerperal fever, 1st, the synochal; 2d, the gastro-bilious; and 3d, the epidemic or contagious puerperal fever. Dr. Churchill observes, "that he (Dr. Douglas) has drawn a marked distinction between ordinary and epidemic puerperal," and as his description serves to illustrate in a measure our author's views on this subject, we make the following extract of his quotation from Dr. Douglas.

"That form of the disease which I arrange under the third head, is really the contagious, or epidemical puerperal fever, and though agreeing with the others in the great leading symptoms, inflammation, pain, tumefaction, and tension of the abdomen, yet differing from them in many material characters. The sensorium here is seldom in any degree disturbed, whereas, in others, it is so frequently, and even sometimes is excited to a high degree of delirium. The pulse here is usually from the moment of attack, soft, weak and yielding, and in frequency often exceeds 160; whereas, in the first species it is full, bounding, and incompressible, and in the second, small, hard and concentrated, and in both moderately frequent. The eye, instead of being suffused with a reddish or yellow tint, as in the others, is here generally pellucid, with dilated pupil; the countenance, instead of being flushed as in the others, is here pale and shrunk, with an indescribable expression of anxiety; an expression altogether so peculiar that the disease could, on many occasions, be pronounced or inferred from the countenance alone. The surface of the body, instead of being, as in the others, of a high pyrexial heat, is here usually soft and clammy, and of heat not above the natural temperature; and not only is the skin cool, with clammy exudation, but the muscles to the impression of the finger, feel soft and flaccid, as if deprived of their vis insita by the influence of the contagion. Indeed, there is such prostration of strength and depression of vital principle from the very onset of the attack, that I must suppose the contagion to act on the human frame, probably through the medium of the nervous system."

The author again quotes Dr. D. as follows:

"The contagious puerperal fever of Dublin is, I venture to pronounce, neither more nor less, than a malignant fever of a typhoid type, accompanied with an erysipelatous inflammation of the peritoneal covering of the stomach, intestines and other abdominal viscera."

The history, or more properly, the sketch of the different epidemics, proceeds from one to the other with great rapidity; that which occurred in 1813, in the northern countries of England, so ably described by the late Dr. Armstrong, is alluded to as closely resembling the Aberdeen and Leeds epidemics, all the cases presented the same incontestible proofs of inflammation, and blood-letting—early and copious blood-letting—seems to have been the only successful mode of treatment. " All who were seized with the disease died,—who were not bled at the beginning." This is in accordance with the recorded statements of Hey and Gordon, and was doubtless excellent practice in those epidemics which displayed a strong inflammatory type.

The experience of Dr. Gooch is next adduced. Dr. Gooch agrees in all the principal points of treatment, but makes, however, a quotation from Dr. Farre, who states that " At the east end of London, not far from the river, this disease proved still more fatal. During the month of March, 1825, one surgeon lost seven, another four cases, in all of which the disease was treated at the instant of its formation by active blood-letting. A physician accoucheur who attended in consultation, told him (Dr. Farre) that, out of thirteen cases eleven died, that *all* which had been bled, died, and that the only two that recovered had not been bled, but were treated with turpentine.

Mr. Labatt's account of the epidemic that prevailed in Dublin in the years 1819–20, is highly interesting, and is well worthy of attentive perusal, both from its intrinsic merits and the high character of the author.

Mr. Labatt used every effort to prevent the spreading of the contagion; the sick patients were separated from the sound ones, scourings, fumigations, and white washings were called in requisition, but all to no purpose, neither cleanliness, nor ventilation, nor the destruction of the utensils and furniture that had been used by those already smitten, seems to have checked the march of the disease, nor lessened its frightful mortality. Dr. Labatt says : " That from sad experience of this epidemic, I am satisfied that the contagion of typhus fever is capable of giving rise to puerperal fever ; that puerperal fever is communicable from one patient to another, and also that it can be carried from the sick by an attendant to women in child-bed who were previously free from disease."

This statement is strongly confirmatory of the opinion that there is a contagious form of puerperal fever. Such, we believe, is the opinion of most physicians of the present day.

An epidemic puerperal fever occurred in Vienna in the year 1819. The patients were attacked on the first, second, or third day after delivery; symptoms of inflammation of the peritoneum and uterus were always present. One patient died in six hours after she was attacked, others in twelve or twenty-four hours.

When the disease presents itself in so malignant a form, one mode of treatment is as good as another, or to speak more correctly, all treatment is equally useless, the powers of life give way at once, and we should imagine a man would as soon think of plunging his lancet into such a patient as he would of bleeding a corpse. "The very rapid putrefaction after death, the dissolved state of the blood, the strikingly soft and tender state of the whole bowels, the heart, lungs, liver, spleen, kidneys, and particularly of the uterus, indicated a colliquative, putrescent condition of the whole system induced by the disease."

Dr. Robert Ferguson's experience is given by one Methor, who states that in the general lying-in hospital, in the years 1835–38, every plan of treatment was tried—bleeding early and copiously, amongst the rest, without producing any good effect, or lessening in the smallest degree the mortality. Seeing that no treatment was of avail, the hospital was closed from May to November. Dr. Ferguson adds, "That the present year, 1838, has exercised an exceedingly fatal influence in every species of fever, all of which were of the low, or typhoid type."

An epidemic prevailed in Paris in the year 1829, in the practice of M. Desormeaux. Mr. Tonnellé, who has described the epidemic, traced the morbid lesions with great care in no fewer than 222 cases. We insert the following condensed summary of M. Tonnellés statements given by the author, not because they contain any pathological novelty, but to show how perfectly they agree with the post mortem observations of our own country.

"In 193 there were traces of peritonitis; in twenty-nine, or about one-eighth there were none.

"In 197 cases, or about nine-tenths, he found morbid lesions in the uterus e. g., simple inflammation of the uterine veins and lymphatics, and softening and putrescence of the uterine parietes.

"In 62 cases the ovaries were inflamed.

"In 90 cases there was inflammation of the veins; in forty of the lymphatics alone.

"In 49 cases the uterus was softened, superficially in 29, deeply in 20.

"In 29 cases there were the usual evidences of pleurisy; in six others an effusion of blood, and in eight of serum into the pleural cavities.

"In 27 cases the lungs were affected, viz., in ten there was pneumonia; in eight, abscess; in four, tubercles; in three, gangrene; in two apoplexy.

"There were purulent collections in the muscles in fourteen cases; in the joints in ten; and in the cellular tissue of the pelvis in six cases.

"Abcess of the liver existed in three cases, and of the pancreas in two cases."

The author adds, that "M. Tonnellé divides puerperal fever into three varieties; the inflammatory, the typhoid, which was the most frequent, and the anomalous or ataxic. "The more active remedies were general bleeding, leeches, ipecacuanha and mercurial salivation." It appears that fully one-third of the cases died.

Next we have the observations of Mr. Ceely of Aylesbury, who has described an epidemic which occurred in that city and its neighborhood in the year 1831, during the prevalence of erysipelas, which exhibited a mild, a phlegmonoid, and a typhoid form, the puerperal seems to have assumed analogous characteristics.

Mr. Ceely says that he had no opportunity of making a post-mortem in the acute cases, from which we presume that they all recovered, which, under active treatment, as far as our experience goes, they generally will. He examined some of the typhoid cases, and found most of the usual appearances, which have been described so often that it would be waste of space and time to repeat them.

"A report of the secondary midwifery institution at Vienna, by Dr. Bartsch, was published in the Lancet, in which it is stated that of 2218 women delivered at that institution between October 15th, 1833, and December 31st, 1834, 175 had puerperal fever, of whom 109 died."

In this report puerperal fever is distinguished from peritonitis and metritis as will be seen from the following quotation.

"The cases of puerperal fever occurred seldom under the form of puerperal peritonitis, but generally as inflammation of the uterine veins, giving rise to the production of puss in these vessels, and the general symptoms accompanying its absorption."

We confess that we are glad to see this distinction made between peritonitis and malignant puerperal fever; it is most rational, and will at once account for the different success of different modes of practice, point out in what cases we ought to bleed without hesitation, and where it would be advisable to use a discriminating judgment, and spare the vital power which is already sinking but too rapidly.

The following quotation is from Dr. Beatty's Report of the Lying-in Hospital, South Cumberland street, Dublin.

"The hospital was visited by this terrible malady twice during the period embraced by the present report. Both attacks took place in the month of January, and at each time erysipelas was raging as an epidemic in the surgical hospitals, and diseases of a typhoid type were very prevalent in the city."

Dr. Beatty lost eight patients out of thirteen. M. Voillemier, Paris, 1838, describes two forms of the disease, the inflammatory and typhoid. The inflammatory form generally yielded to active antiphlogistic treatment, though occasionally it terminated fatally. In the typhoid form the patients rapidly sank at the end of a few days or hours. There was no regularity in either lochia or milk. In a few cases M. Voillemier thought he could trace the origin of the disease to contagion.

"Epidemics of puerperal fever occurred at Rennes in 1842 and 1844, and have been described by M. Betral. The lymphatics were principally implicated, the veins being unaffected. The disease sometimes terminated in forty hours, but generally not before the fifth day. The mortality in the first epidemic was twenty out of twenty-four, and in the second, twenty out of twenty-two. There were purulent deposite in the lungs."

The disease also appeared in the Westminster Lying-in Hospital in the year 1842, an account of which has been given by M. Buddy.

A slight sketch of some other epidemics is then given, but as we labor under the apprehension of making our article too long, we omit them, and proceed to give an extract by our author, from Dr. McClintock, Rotunda Lying-in Hospital, 1845. The Dr. thus enumerates the peculiarities of the outbreak:

"1st. The very sudden and unexpected manner in which the epidemic appeared, without any of those precursory warnings which have usually preceded its invasion. 2d. The remarkable circumstance, that of the fourteen children of the women attacked, five died: one of rapid trismus, one of erysipelas, and three of convulsions. 3d. That out of the ten fatal cases, nine were examined post mortem, which examination revealed the most extensive morbid appearances, quite adequate to account for death. 4th. During the same period that puerperal fever was in our wards, erysipelas was very prevalent in some of the surgical hospitals throughout our city. 5th. It is worthy of remark what a small detraction of blood was sufficient to bring on syncope in this epidemic. Nearly every case was bled as soon as the system had rallied from the rigor; but only one woman (who recovered) bore the loss of so much as fifteen ounces, whilst from six to eight ounces was about the average."

There are some other epidemics historically mentioned, but we presume that enough has been given to show the nature, and even to convey some slight idea of the merits of the article which cannot be appreciated too highly; though short, it must have cost the author much labour and research; and though he admits that the list is in all probability imperfect, yet we agree with him cordially that, so far, it is more complete than any with which we are acquainted. We recommend the careful perusal of it to all practitioners, along with the tables of the various epidemics, which the author has been at the pains to give us, in order to render it more intelligible, but which for obvious reasons could not be inserted here.

Having finished his summary, the author concludes with some few observations, rather as suggestions, to induce his readers to follow up the subject than as absolute inferences. They appear to us to be so judicious, that we shall make no apology for giving some of them, and as we before gave our readers a promise to that effect, we shall give them in Dr. Churchill's own words.

"I would remark then, in the first place, that there appears some especial connection between the epidemics of puerperal fever, and lying-in hospitals. I do not mean exactly to assert that these epidemics always originate with, and are kept up by, the hospitals; but I refer to the fact that we have no record of any epidemic independent of them in early times. The first in France, England and Ireland, occurred in the Hotel Dieu of the former, and the lying-in hospitals of the latter countries, and though our earlier authors allude to inflammation of the womb, &c., occurring in child-bed, they make no mention of its prevailing extensively as an epidemic."

The author then alludes to the almost universally admitted fact, that puerperal fever is always, or almost always, connected with local diseases, but adds that Dr. Copland, in an excellent article, has denied the universal presence of inflammation in the malady, and states that in one epidemic the only pathological characteristics observable were a remarkable alteration of the blood, general lacerability of the tissues, or loss of their vital cohesion soon after death. He adds, however, that such cases are rare.

We proceed with the author's remarks :

"I repeat my conviction that there are few if any cases of puerperal fever without local disease of the organs employed in parturition or the neighboring tissues; but are we therefore justified in asserting with Dr. Lee that puerperal fever is simply a local affection?

"I have latterly seen reason to doubt the truth of the view I formerly took, which was in accordance with that of Dr. Lee, and though I would wish to express myself cautiously and guardedly, I must honestly avow, that whilst I fully admit the existence of local disease, I do think that epidemic puerperal fever is something more than that, although I may not be able to define exactly what it is.

"We should be justified in this supposition I think on several grounds. First, the very remarkable variety of opinion as to its nature, would go far to prove that it cannot be the simple local disease Dr. Lee believes. For example, by some it is regarded as an inflammation of the uterus; by others inflammation of the omentum and intestines; by a third party as peritonitis; by a fourth erysipelatous inflammation; by a fifth and sixth as a fever sui generis, or with biliary disorder; by a seventh as a disease of a putrid character, &c. Such different views are hardly reconcilable with the notion of a simple inflammation."

We have long been of precisely the author's opinion, but he does not appear to have any very distinctly defined idea on the subject, and indeed the difference of opinion is so great, that it is hard for a man who seeks truth and not the gratification of his vanity, by the exemplification of some favorite dogma, to bring his mind to a conclusion. The arguments remind us forcibly of the history of the cameleon. The author proceeds with the following remarks.

"Then again look at the prevailing characters of different epidemics, and see how varied they are; in one the lochia are suppressed, in another they are profuse, in a third unaltered ; diarrhœa is common in one epidemic, constipation in another ; typhoid symptoms in one, ordinary fever in another. And as to remedies we find even a greater diversity: one very high authority recommends saline purgatives; another loses all his patients until he bleeds largely at the commencement; another loses those who are so bled. Calomel is the universal remedy in one epidemic, opium in another.

"Lastly, let any one compare a case of simple inflammation of the womb or peritoneum in child-bed, with a case of epidemic puerperal fever, their symptoms, course, and the effect of remedies, and I do not think that a doubt will remain upon his mind that, although the latter is a local disease, it is not exclusively so."

The author then enquires, what more is it than a local disease? discusses the peculiar effects of uterine phlebitis, states Mr. John Hunter's opinion that phlebitis destroys life by the extension of inflammation to the heart, a position which Dr. Arnott's investigations disproved, showing that it was probably owing to an alteration in the quality of the blood. "M. Bouillaud, in 1825, attributed the typhoid symptoms in phlebitis to the mixture of pus with the blood." Analagous results have been produced by injecting putrid matter into the system, and "Guthrie's descriptions," says Dr. Churchill, "of the characteristics of irritative phlebitis symptoms, &c., are very like those of puerperal fever."

Many authorities are quoted in support of the plausibility of the position that puerperal fever, occurring as an epidemic, is neither more nor less than typhus, modified by the peculiar condition of the female at the parturient time, and it is natural enough to suppose that those organs and their investments more immediately concerned in the process of labor, should, be most liable to be affected by the vitiated condition of the general system, and take on an unhealthy inflammatory action, producing all that local mischief which Dr. Lee and others have maintained is the whole disease.

When we take into consideration the fact that typhus and erysipelas frequently appear together, and during the same atmospheric conditions, the supposition that the local affection is of an erysipelatous character, is extremely plausible.

The following quotation from Mr. Nunnelly, is corroborative of the author's opinion on this subject.

"It is highly probable, if not certain, that there is some change produced in the state of the blood, which change may depend upon alterations we are unable at present to appreciate, but which it is likely occur in many tissues, and may thus affect the mass of the blood more or less quickly, and to a greater or less extent, according to the influence they have upon, and the connection they have with the blood in a state of health."

The important subject of contagion is then discussed—various high authorities, both for and against the contagiousness of child-bed fever, are quoted; the author does not seem to be fully satisfied on this subject, but all that he says has been before the medical public so often, that we think it unnecessary to trouble our readers by calling their attention particularly to his opinions. They are, nevertheless, worthy of great respect, and may be read with advantage. One observation we quote as follows:

"As in all cases where a disease is epidemic, it is and must be a difficult thing to decide as to the contagiousness of puerperal fever; still I confess that the facts would lead me to the inference that, at least, it is communicable from a woman laboring under it to others in the same ward."

These remarks naturally lead to the consideration whether puerperal fever can or cannot be conveyed by a third party in health, from a person laboring under it to another person in child-bed. This is a subject of momentous importance to the practitioner of midwifery, and one which we heartily wish could be determinately settled; if it be as some have thought, (Gordon amongst the rest) that the medical attendant himself carries death into the chamber of his confiding patient, every conscientious man who meets with a case of puerperal fever, of a typhoid character, must be subjected to a fearful anxiety, for he must either continue his practice at the conscious hazard of destroying those entrusted to his care, or he must impoverish himself by abandoning his professional duties, a hard alternative to a man dependent for his living on such exertions.

Dr. Churchill does not seem to have settled this question either way to his own satisfaction, and certainly does not satisfy his readers; he says:

"So far as the weight of opinion goes, it is in favor of contagion, but I think we are scarcely yet in a position to speak quite positively."

After quoting many anecdotes of cases, in which the authors (men of high repute) assert that in their judgment the epidemic was conveyed by the attending physicians from the sick to healthy women, he makes the following commentary : "'Post hoc' is not always 'propter hoc,' however, and we must not forget that puerperal fever was epidemic at the time." This remark has particular reference to some cases which occurred in Edinburgh in 1821–2.

The author winds up with the following remarks:

"The evidence and proofs thus adduced are of extreme importance, and I fear we must conclude, however reluctantly, in favor, not only of the contagiousness of puerperal fever, but of the possibility of its contagion being carried by an intermediate party. This makes the practice of midwifery doubly distressing during the prevalence of an epidemic, and ought deeply to impress us with the necessity of the utmost care and caution."

The article closes with the following quotation from Dr. Copland.

"An obstetric physician should not make an autopsy of a case of puerperal fever or of erysipelas, or of peritonitis, or of diffusive inflammation of the cellular tissue, or of the disease occasioned by the necroscopic poison; nor even attend, dress, nor visit any of such cases without observing the utmost precaution with regard to ablution and change of clothing, and allowing two or three days to elapse between such attendance and midwifery engagements, or visits to puerperal females."

We have thus traced, as connectedly as our space would admit of, the original portion of Dr. Churchill's book, viz., the historical sketch of puerperal fever. The essays, though never before published together, have been in the hands of the profession many years, and have been reviewed and commented on so frequently, that further notice of them would be

"As tedious as a thrice told tale
Vexing the dull ear of a drowsy man."

We will only add that the work as a book of reference is invaluable, and ought to form a part of every medical library.

